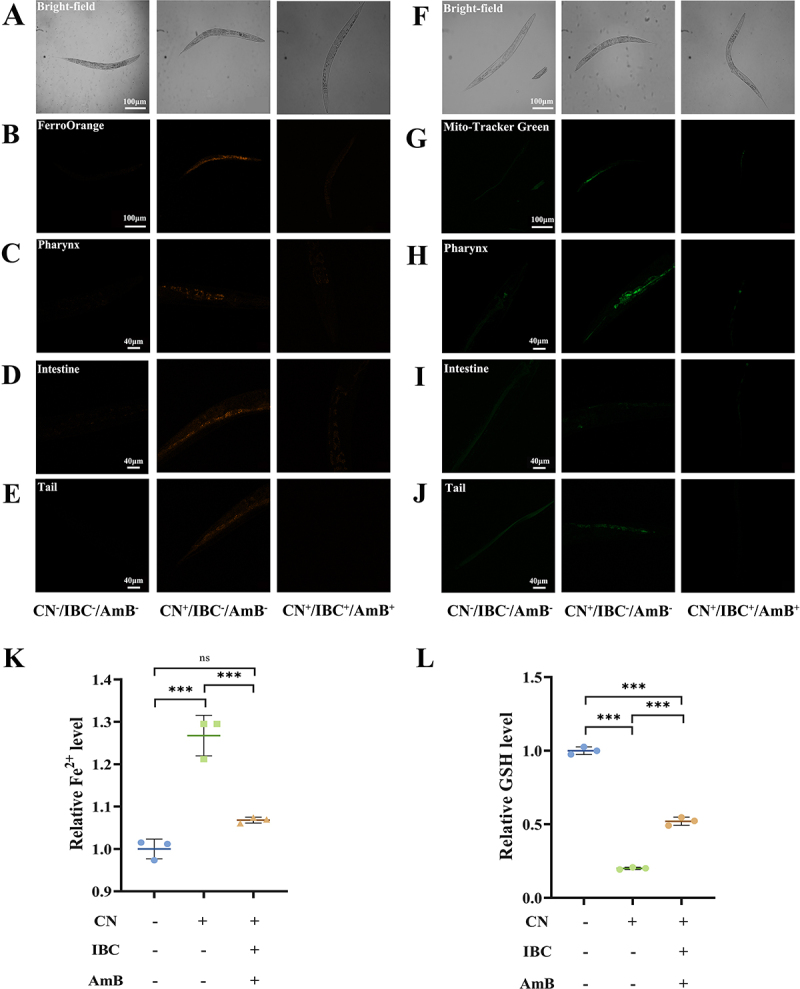# Correction

**DOI:** 10.1080/21505594.2025.2553472

**Published:** 2025-09-01

**Article title**: Combination of isobavachalcone and amphotericin B has antifungal effect against Cryptococcus neoformans and protects host tissue damage by inhibiting ferroptosis

**Authors**: Weidong Qian, Na Liu, Jiaxing Lu, Qiming Liu, Si Chen, and Ting Wang

**Journal**: *VIRULENCE*

**DOI**: http://dx.doi.org/10.1080/21505594.2025.2543981

When the article was published earlier, the figure 6 correction was inadvertently missed by the publisher. This has now been corrected in the original article.

**Figure:**